# Functional and Aesthetic Outcomes of Post-Mohs Nasal Reconstruction

**DOI:** 10.3390/cmtr18010017

**Published:** 2025-02-20

**Authors:** Nora A. Alexander, Kwasi Enin, Jenny Ji, Emily Spataro

**Affiliations:** Division of Facial Plastic and Reconstructive Surgery, Department of Otolaryngology—Head & Neck Surgery, Washington University School of Medicine in St. Louis, St. Louis, MO 63141, USA; j.jenny@wustl.edu (J.J.); emily.spataro@wustl.edu (E.S.)

**Keywords:** Mohs micrographic surgery, facial reconstruction, outcomes, complications

## Abstract

Background: Similar to patients undergoing rhinoplasty, patients undergoing post-Mohs micrographic surgery (MMS) nasal reconstruction are concerned with both nasal form and function. Objectives: The objectives were to identify patient, defect, and surgical characteristics associated with post-MMS nasal reconstruction outcomes. Methods & Materials: A retrospective single-institution cohort study was conducted of post-MMS nasal reconstruction surgeries occurring between 2015 and 2020. Patient, defect, and surgical details were collected along with nasal aesthetic and functional outcomes. Multivariable logistic regressions were used to analyze data. Results: A total of 167 nasal MMS defects among 160 patients met the inclusion criteria. The median age was 66 years, and 70% were female. A poor aesthetic outcome (*n* = 20, 12.0%) was associated with diabetes (aOR 6.277, 95% CI 2.193–17.965). Post-operative nasal obstruction (*n* = 17, 10.2%) was associated with obesity (aOR 20.976, 95% CI 2.406–182.845) and major revision surgery (aOR 12.192, 95% CI 2.838–52.382). Conclusion: Post-MMS nasal reconstruction aesthetic and functional outcomes are associated with obesity, diabetes, and revision surgery. Improved standardization of functional and cosmetic outcomes is important to better understand these results in the future.

## 1. Introduction

Nonmelanoma skin cancer (NMSC) is the most common malignancy worldwide [1] with an estimated prevalence of 5 million cases and 3 million people undergoing treatment yearly within the United States [1]. Mohs micrographic surgery (MMS) is a specialized technique employed by dermatologists to remove skin cancer and assess margins, particularly those that are located in important areas such as the nose. Among facial Mohs micrographic reconstructions, the nasal subsite is the most frequent defect subsite, with literature reports ranging from 50–60%+ [2,3].

As the nose is an important facial structure that plays a critical role in breathing, smell and its synergy with taste, and overall facial aesthetics, successful reconstruction of nasal defects relies on minimizing both cosmetic and functional deficits. Many patient factors can impact wound healing, including diabetes, obesity, use of steroids/immunomodulators, smoking, and prior radiation [4]. In the facial reconstructive literature, data conflict on which medical comorbidities, defects, or surgical factors are associated with reconstructive complications.

A unique aspect of nasal reconstruction is the risk of nasal obstruction. Nasal obstruction can occur for various reasons, such as the formation of scar tissue, septal deviation, or the collapse of nasal valves [5]. Obstruction can result in significant quality of life impairments [6,7]. Rates of airway collapse following nasal reconstruction may depend on the reconstruction subsite, size, and grafting materials, with rates reported in the literature as low as 1.3% in alar and sidewall cartilage-grafted reconstructions and as high as 78% in alar reconstructions with melolabial interpolated flaps without cartilage support [8,9]. Successful nasal reconstruction should aim to prevent or minimize the risk of obstruction by ensuring adequate airflow and preserving structural integrity of the nasal valve. Various patient factors, such as smoking, prior radiation, and medical comorbidities, can increase the risk of obstruction and should also be considered during the reconstruction process. The choice of reconstructive method may also increase the risk of obstruction dependent on the flap pedicle size, excess tissue bulk, or extent of scar contracture, but this association is also disputed [10,11,12].

Another primary concern in nasal reconstruction is achieving patient-desired aesthetics. The nose is a critical facial feature that significantly contributes to one’s overall appearance, and any cosmetic deformity can be distressing for the patient [13]. It has been shown in the prior literature that large, central facial defects, such as those on the nose, garner more attentional distraction than smaller, more peripheral defects [14]. Achieving the desired aesthetic outcome can be particularly challenging in nasal reconstruction due to the variety of contour changes, skin thickness and texture differences, and individual patient anatomical features and proportions at each various nasal subsite. These considerations have intricate relationships with the choice of reconstructive technique, as well as post-operative wound healing and scar management.

While there is a considerable amount of research on the safety and efficacy of MMS and facial reconstruction, further investigation is needed to better understand the patient, defect, and surgical factors that can impact outcomes of post-Mohs micrographic surgery nasal reconstruction. This study aims to assess these factors and their associations with aesthetic and functional outcomes in patients undergoing post-MMS nasal reconstruction.

## 2. Methods

A retrospective single-institution cohort study of post-MMS nasal reconstructions occurring between 1 January 2015 and 31 December 2020 was conducted at Washington University School of Medicine in the Department of Otolaryngology—Head and Neck Surgery. The study was approved by the Washington University Institutional Review Board. Inclusion criteria included all patients undergoing reconstruction of a nasal defect after MMS. MMS occurred either within or outside the institution. Exclusion criteria included if the date of MMS was unknown, if a cancer excision method other than MMS was undertaken (such as wide local excision), and the melanoma skin cancer type as patients did not undergo standard MMS.

The collected data included patient demographics and comorbidities, surgical factors such as defect characteristics and reconstruction details and time from MMS to repair, as well as postoperative outcomes such as complications, minor and major revisions, and patient- and physician-reported nasal obstruction and aesthetics. Documented complications including dehiscence, skin sloughing, skin necrosis, and flap partial or full failure were collectively categorized as poor wound healing; other complications collected included bleeding, infection, and patient-reported post-operative new or worsened nasal obstruction. A minor revision was performed in the office and included procedures such as debridement of skin sloughing, drainage of small seromas, scar revision, and minor flap debulking. Major revisions were procedures that needed to be performed in the operating room, such as drainage of a large hematoma, insetting a new flap in the case of flap failure, or more major flap debulking/repositioning that could not be performed in the office. Patient- and physician-reported outcomes were identified on a per-case basis. Documentation in the medical records of prolonged scar management (greater than 6 months to 1 year), patient expression of significant concern with their appearance requesting revision/intervention, physical exam findings of prominent scar, scar hypertrophy, and/or contour abnormality of the nose were recorded as poor aesthetic outcomes. Analysis of poor aesthetic outcome included both those defined by the patient and/or the physician. Documentation in the medical record of patient-reported nasal obstruction, defined as new or worsened from baseline after their nasal reconstructive surgery, was also collected.

Statistical analysis included descriptive statistics and multivariable logistical regression to assess the patient, defect, and procedural characteristics associated with nasal obstruction and poor aesthetic outcome. To avoid overfitting the regression model [13], univariate logistic regressions were performed first to identify potentially significant variables (*p* < 0.05). Those variables were then included in a multivariable logistic regression. This analysis allowed identification of the independent weight each factor had on poor aesthetic outcome and nasal obstruction, reducing the impacts of other potential colinear variables (e.g., large, full thickness defects were all likely reconstructed with forehead or melolabial flaps). All data analysis was performed with IBM SPSS Statistics, version 28 (Armonk, NY, USA).

## 3. Results

Included for analysis were 167 nasal MMS defects among 160 patients. Demographic data and their univariate logistic regression associations with poor aesthetic outcomes or nasal airway obstruction are summarized in Table 1. The median age of the patients was 66 years (range 26–95). Most patients were female (71.3%, n = 119). Comorbidity data included 55.7% (n = 93) of patients with hypertension, 16.2% (n = 27) with diabetes, 28.1% (n = 47) taking anticoagulation, and 19.8% (n = 33) with another comorbidity. Most patients (52.7%, n = 88) were never smokers, whereas 34.7% (n = 58) were former smokers, and 12.6% (n = 21) were current smokers. Most defects (92.8%, n = 155) were due to basal cell carcinoma (BCC), and 7.2% were due to squamous cell carcinoma (SCC).

Defect and reconstruction characteristics and their univariate logistic regression associations with poor aesthetic outcomes or nasal airway obstruction are summarized in Table 2. Most cases involved the nasal ala (51.5%, n = 86) and/or tip (41.9%, n = 70); 15.0% (n = 25) of cases involved multiple nasal subsites. In 25 cases, the defect involved multiple subsites but from a single defect (i.e., the defect encompassed the nasal tip, dorsum, and ala). Thus, the total number of subsites affected was 228, and 25 of the 167 cases had 2 or more subsites affected. Most defects (48.5%, n = 81) were reconstructed with local soft tissue reconstruction, of which 25 (15.0%) utilized primary closure, 20 (12.0%) utilized bilobed flaps, 22 (13.2%) utilized two-stage paramedian forehead flaps, 1 (0.6%) utilized one-stage paramedian flaps, 19 (11.4%) utilized single-stage melolabial flaps, 20 (12.0%) utilized two-stage melolabial flaps, and 50 (29.9%) utilized other local tissue advancement methods. Sixty (35.9%) also required cartilage or composite grafting. Again, a subset of patients underwent one or more reconstructive methods. Overall, there were 29 (17.4%) complications and eight (4.8%) cases of infection, and 17 (10.2%) cases had nasal obstruction post-operatively. During the follow-up period, 25 (15.0%) minor in-office revisions were performed, and 18 (10.8%) major revisions in the operating room were performed.

Table 3 summarizes the results of the multivariable logistic regression analyses with nasal airway obstruction or poor aesthetic outcomes, which included all univariate associations that were statistically significant at an alpha level of 0.05 from Table 1 and Table 2. For nasal airway obstruction, univariate factors of obesity (odds ratio (OR) 9.867, 95% confidence interval (CI) 1.295–75.174), full thickness defects (OR 3.338, 95% CI 1.163–9.482), local skin and soft tissue reconstruction (OR 0.284, 95% CI 0.089–0.911), two-staged paramedian forehead flaps (OR 6.300, 95% CI 2.090–18.991), and major revision surgery (OR 17.625, 95% CI 5.489–56.598) were included in the multivariate analysis. Of these factors, obesity (adjusted odds ratio (aOR) 20.976, 95% CI 2.406–182.845) and major revision surgery (aOR 15.218, 95% CI 3.358–68.954) demonstrated statistically significant associations with nasal airway obstruction on multivariate analysis.

When looking at poor aesthetic outcomes, univariate analyses yielded the following significant associations: diabetes (OR 5.864, 95% CI 2.137–16.092), obesity (OR 8.056, 95% CI 1.068–60.740), and nasal airway obstruction (OR 3.750 95% CI 1.162–12.103). Of these factors, only diabetes (aOR 6.277, 95% CI 2.193–17.965) held a statistically significant association on multivariate analysis.

## 4. Discussion

The primary outcomes of this study included that about 10% of patients experienced nasal obstruction after reconstruction, and 12% of patients had poor aesthetic outcomes. Additionally, 17.4% of nasal reconstructions had some form of complication, with 15.0% requiring minor revisions in the office, and 10.8% requiring major revisions in the OR. These findings are within the commonly reported range of 1–19% [10] and reflect the complexity of Mohs reconstructions centered on the nose, even in the experienced hands of facial plastic surgeons.

Obesity, full thickness nasal defects, paramedian forehead flaps, and major revision surgery were associated with greater frequency of nasal airway obstruction on univariate analysis. These factors point to magnified severity and complexity of the reconstruction and increased disturbance of the normal facial anatomy. Interpolated flaps such as the paramedian forehead flap are used in the approach to large defects, where local tissue advancement is not feasible, or more robustly vascularized tissue to the wound bed is necessary [15]. Risks, however, include distal tip necrosis, bulkiness of the flap, and other contour or functional deformities, as well as poor scarring of both the recipient and donor site and facial asymmetry [16]. We hypothesize that the bulkiness of the paramedian flap contributes to nasal obstruction, as would increased adiposity within the subcutaneous layer in patients affected by obesity. Surgeons may have also raised thicker flaps on obese and/or diabetic patients due to the association between metabolic disorders and poor wound healing from vascular compromise and nutritional deficits [17]. Full thickness defects and paramedian forehead flap reconstruction were no longer statistically significant on multivariable analysis, as they were likely co-linear variables. The association of major revision surgery with nasal airway obstruction is intuitive, as the functional deficit and decreased quality of life associated with nasal airway obstruction makes it a common indication for revision surgery in Mohs reconstruction or rhinoplasty [18].

Poor aesthetic outcomes, whether reported by patients or physicians, were associated with obesity, diabetes, and nasal airway obstruction. As previously discussed, obesity and diabetes can be associated with poor wound healing due to vascular compromise and nutritional deficits. The literature has conflicting reports regarding reconstructive surgeries and the impact of obesity as a risk factor for complications and its association with overall patient satisfaction [19]. The likely contributing factor is increased impairment of wound healing, which is more frequently observed in patients with obesity. It also stands to reason that patients affected by nasal airway obstruction had more extensive defects requiring the use of bulky advancement or interpolated flaps, which would involve more disturbance of normal anatomy than primary closure, affecting aesthetic results as well.

The results of multivariate analysis revealed a limited selection of significant associations: nasal airway obstruction was associated with obesity and major revision procedures, while poor aesthetic outcomes were only associated with diabetes. These results suggest that both nasal obstruction and poor aesthetic outcomes are likely inter-related and correlate with bulkier reconstructions, whether that be due to large, full thickness defects or patient factors such as diabetes and obesity, which may lead surgeon to choose better vascularized flaps such as the forehead flap or raise thicker flaps during reconstructive procedures to minimize poor vascularity to the flap. When safe to do so, we advocate raising a thinner flap, such as in the subcutaneous plane for forehead flap reconstruction, or potentially staging these for intermittent debulking procedures if deemed not safe to do this at the first stage. In patients for whom there is concern that flap debulking would compromise flap viability (those with diabetes or obesity), then using these data would be helpful in counseling them on the likelihood they may need revision procedures in the future for both nasal obstruction and nasal aesthetics.

There are limitations to this analysis. Namely, there was no standardized outcome measure used to assess nasal aesthetics, and surrogates in the medical record related to poor nasal contour, scarring, or need for revision were used. While there are a variety of outcome measures for nasal reconstruction, there is no single best questionnaire or objective tool that has high correlation with long-term patient symptomatology and psychological satisfaction [6,20,21,22,23]. Current options to measure nasal obstruction and related complications following reconstruction include patient-reported questionnaires, as well as objective assessments such as rhinomanometry or nasal peak inspiratory flow, among others [20,21,22]. These instruments, however, are validated in patients undergoing nasal surgery to address structural issues in the nose (septoplasty or functional rhinoplasty) and not reconstruction for skin cancer deficits. Assessment of these outcomes for nasal reconstruction patients would be enhanced by validation in this patient population. Similarly, while patient-reported outcome measures can be used to evaluate aesthetic outcomes after nasal reconstruction, no standardized scale has been validated in this patient population [24].

While this study provides information regarding factors associated with complications and revision surgeries in functional and cosmetic outcomes of nasal reconstruction, other limitations included that several patients were lost to follow-up or had very few follow-up visits. Importantly, many outcome variables were not reliably charted, especially regarding patient-reported assessment of outcomes. Other limitations of this study include its retrospective nature, as well as only having data from a single academic institution.

## 5. Conclusions

Among a cohort of patients undergoing nasal reconstruction after Mohs micrographic surgery, the rate of complications was comparable to those reported in the literature; obesity, full thickness defects, paramedian forehead flaps, and major revision surgery were associated with nasal airway obstruction, while obesity, diabetes, and nasal airway obstruction were associated with increased risk of poor aesthetic outcomes. This data provides value preoperative and postoperative assessment information for patients and physicians. Future studies assessing patient-reported outcomes through validated scales and measures would complement this research.

## Figures and Tables

**Table 1:** Study population demographics and patient characteristics with univariate logistic regressions.

Demographics	Total Defects, No. (%) (N = 167)	Patients with Poor Aesthetic Outcomes, No (%) (N = 20)	Univariate OR (95% CI)	Patients with Nasal Airway Obstruction, No. (%) (N = 17)	Univariate OR (95% CI)
Age					
18–49	13 (7.8)	3 (15.0)	4.20 (0.814–21.678)	0 (0.0)	0
50–59	40 (24.0)	5 (25.0)	2.00 (0.503–7.957)	7 (41.2)	2.970 (0.808–10.914)
60–69	54 (32.3)	8 (40.0)	2.435 (0.689–8.601)	6 (35.3)	1.750 (0.466–6.568)
≥70	60 (35.9)	4 (20.0)	ref	4 (23.5)	ref
Sex					
Male	48 (28.7)	5 (25.0)	ref	3 (17.6)	ref
Female	119 (71.3)	15 (75.0)	1.240 (0.424–3.626)	14 (82.4)	2.000 (0.548–7.301)
Comorbidities					
Hypertension	93 (55.7)	13 (65.0)	1.555 (0.587–4.121)	10 (58.8)	1.153 (0.417–3.192)
Diabetes	27 (16.2)	9 (45.0)	5.864 (2.137–16.092) ^a^	3 (17.6)	1.125 (0.300–4.216)
Obesity	4 (2.4)	2 (10.0)	8.056 (1.068–60.740) ^a^	2 (11.8)	9.867 (1.295–75.174) ^a^
Anti-coagulation	47 (28.1)	6 (30.0)	1.108 (0.399–3.079)	5 (29.4)	1.071 (0.356–3.227)
Immune modulation	21 (12.6)	1 (5.0)	0.334 (0.042–2.636)	1 (5.9)	0.406 (0.051–3.234)
History of radiation therapy	10 (6.0)	0 (0.0)	0	0 (0.0)	0
Other	33 (19.8)	4 (20.0)	1.017 (0.316–3.273)	6 (35.3)	2.485 (0.845–7.305)
None	43 (25.7)	4 (20.)	0.692 (0.218–2.198)	2 (11.8)	0.354 (0.078–1.618)
Smoking history					
Current	21 (12.6)	10 (50.0)	0.821 (0.166–4.062)	9 (52.9)	0.429 (0.052–3.669)
Former	58 (34.7)	8 (40.0)	1.248 (0.461–3.376)	7 (41.2)	1.205 (0.422–3.438)
Never	88 (52.7)	2 (10.0)	ref	1 (5.9)	ref
Type of cancer					
Basal cell carcinoma	155 (92.8)	20 (100.0)	<10^−8^	16 (94.1)	0.790 (0.096–6.524)
Squamous cell carcinoma	12 (7.2)	0 (0.0)	ref	1 (5.9)	ref

^a^*p* < 0.05.

**Table 2:** Defect and reconstruction characteristics with univariate logistic regressions.

Demographics	Total Defects No. (%) (N = 167)	Poor Aesthetic Outcomes No (%) (N = 20)	Univariate OR (95% CI)	Nasal Obstruction No (%) (N = 17)	Univariate OR (95% CI)
Nasal subsite breakdown					
Dorsum	24 (14.4)	1 (5.0)	0.284 (0.036–2.225)	0 (0.0)	0
Sidewall	45 (26.9)	4 (20.0)	0.646 (0.204–2.048)	5 (29.4)	1.146 (0.380–3.457)
Ala	86 (51.5)	8 (40.0)	0.590 (0.228–1.527)	12 (70.6)	2.465 (0.828–7.341)
Columella	3 (1.8)	0 (0.0)	0	0 (0.0)	0
Tip	70 (41.9)	12 (60.0)	2.302 (0.887–5.974)	8 (47.1)	1.262 (0.461–3.451)
Area of defect (cm^2^) ^c^					
0–1	35 (21.0)	5 (25.0)	2.667 (0.286–24.827)	2 (11.8)	0.455 (0.058–3.541)
1.1–2.25	49 (29.3)	5 (25.0)	1.818 (0.197–16.774)	4 (23.5)	0.667 (0.111–4.014)
2.26–10	63 (37.7)	9 (45.0)	2.667 (0.314–22.665)	9 (52.9)	1.250 (0.244–6.415)
>10	17 (10.2)	1 (5.0)	ref	2 (11.8)	ref
Nasal defect depth					
Skin only	95 (56.9)	12 (60.0)	1.157 (0.446–2.997)	9 (52.9)	0.837 (0.306–2.289)
Skin and cartilage	39 (23.4)	5 (25.0)	1.108 (0.375–3.270)	1 (5.9)	0.184 (0.024–1.436)
Full thickness	33 (19.8)	3 (15.0)	0.688 (0.189–2.503)	7 (41.2)	3.338 (1.163–9.482) ^a^
Type of tissue Reconstruction					
Local skin + soft tissue	81 (48.5)	8 (40.0)	0.676 (0.261–1.750)	4 (23.5)	0.284 (0.089–0.911) ^a^
Composite graft	18 (10.8)	2 (10.0)	0.910 (0.193–4.288)	1 (5.9)	0.489 (0.061–3.923)
Cartilage and local tissue Reconstruction	42 (25.1)	3 (15.0)	0.489 (0.136–1.759)	9 (52.9)	3.989 (1.427–11.147)
Split thickness skin graft	7 (4.2)	2 (10.0)	3.156 (0.570–17.474)	0 (0.0)	0
Full thickness skin graft	37 (22.2)	6 (30.0)	1.604 (0.570–4.516)	5 (29.4)	1.536 (0.504–4.681)
Type of local soft tissue Reconstruction					
Primary closure	25 (15.0)	2 (10.0)	0.599 (0.130–2.759)	0 (0.0)	0
Secondary closure	0 (0.0)	0 (0.0)	0	0 (0.0)	0
Bilobe flap	20 (12.0)	2 (10.0)	0.796 (0.170–3.721)	2 (11.8)	0.978 (0.206–4.632)
Paramedian forehead flap— 1 stage	1 (0.6)	0 (0.0)	0	0 (0.0)	0
Paramedian forehead flap— 2 stage	22 (13.2)	3 (15.0)	1.189 (0.318–4.444)	7 (41.2)	6.300 (2.090–18.991) ^a^
Melolabial flap— 1 stage	19 (11.4)	2 (10.0)	0.850 (0.181–3.987)	3 (17.6)	1.795 (0.465–6.926)
Melolabial flap— 2 stage	20 (12.0)	2 (10.0)	0.796 (0.170–3.721)	1 (5.9)	0.431 (0.054–3.438)
Rhomboid flap	1 (0.6)	0 (0.0)	0	0 (0.0)	0
VY advancement flap	5 (3.0)	0 (0.0)	0	0 (0.0)	0
Other local tissue Advancement flap	50 (29.9)	4 (20.0)	0.567 (0.179–1.790)	4 (23.5)	0.718 (0.222–2.322)
Time between MMS and reconstruction					
0–1 day	104 (62.3)	10 (50.0)	ref	11 (64.7)	
2–7 days	42 (25.1)	5 (25.0)	1.270 (0.407–3.968)	2 (11.8)	0.423 (0.090–1.995)
8–14 days	13 (7.8)	3 (15.0)	2.820 (0.664–11.969)	1 (5.9)	0.705 (0.083–5.950)
>14 days	8 (4.8)	2 (10.0)	3.133 (0.557–17.639)	3 (17.6)	5.073 (1.064–24.184) ^a^
Complications					
Any	29 (17.4)	3 (15.0)	0.821 (0.224–3.009)	2 (11.8)	0.607 (0.131–2.814)
Infection	8 (4.8)	1 (5.0)	1.053 (0.123–9.031)	1 (5.9)	1.277 (0.148–11.050)
Poor wound healing	20 (12.0)	2 (10.0)	0.796 (0.170–3.721)	1 (5.9)	0.431 (0.054–3.438)
Nasal obstruction	17 (10.2)	5 (25.0)	3.750 (1.162–12.103) ^a^	-	-
Intervention/revision					
Minor intervention/revision	25 (15.0)	6 (30.0)	2.887 (0.989–8.425)	3 (17.6)	1.247 (0.331–4.697)
Major intervention/revision	18 (10.8)	4 (20.0)	2.375 (0.697–8.095)	9 (52.9)	17.625 (5.489–56.598) ^a^

^a^*p* < 0.05; Some patients had defects that spanned more than one subsite, received more than one type of reconstruction, had more than one type of complication, and/or received more than one intervention; ^c^ Two defects did not have recorded sizes.

**Table 3:** Multivariable logistic regression model for factors associated with key complications post-MMS nasal reconstruction.

Variable	Poor Aesthetic Outcome Adjusted Odds Ratio (95% CI)	Nasal Airway Obstruction Adjusted Odds Ratio (95% CI)
Comorbidities		
Obesity	5.221 (0.589–46.296)	20.976 (2.406–182.845) ^a^
Diabetes	6.277 (2.193–17.965) ^a^	-
Nasal defect thickness		
Full	-	1.390 (0.296–6.483)
Reconstruction type		
Local skin and soft tissue	-	0.572 (0.142–2.301)
Type of reconstruction		
Paramedian forehead, two-stage	-	1.477 (0.248–8.792)
Complications		
Nasal airway obstruction	3.479 (0.945–12.815)	-
Revision surgery		
Major	-	15.218 (3.358–68.954) ^a^

^a^*p* < 0.05.

## Data Availability

Data is unavailable due to privacy restrictions.
